# PD33 - Drug Reaction and Eosinophilia with Systemic Symptoms (DRESS): a 10-year review in a pediatric population

**DOI:** 10.1186/2045-7022-4-S1-P33

**Published:** 2014-02-28

**Authors:** Sharon Wong, Mark Koh

**Affiliations:** 1 Department of Pediatric Medicine, KK Hospital, Singapore; 2 Department of Dermatology, KK Hospital, Singapore

## Background

DRESS, also known as drug-induced hypersensitivity syndrome, is a rare but severe and potentially fatal adverse drug reaction. This is a review of the epidemiology and management of DRESS in Asian Singaporean children.

## Method

10-year retrospective study of patients admitted for DRESS. Cases were identified by relevant ICD codes from inpatient records.

## Results

7 patients with DRESS were identified. Patients ranged from 8 to 16 years old at the time of presentation. Inciting drugs were: Bactrim (3 cases), augmentin (1), carbamazepine (1), phenobarbitone (1), sulfasalazine (1), traditional chinese medication (1). Symptom onset ranged from 10 days to 6 weeks from the start of the inciting drug.

All patients had high fever and generalized pruritic exanthematous rash. Two patients also had desquamative rashes, and one patient had purpuric papules. 4 patients had facial oedema, 4 patients had oral mucositis. Most patients had lymphadenopathy and hepatomegaly.

6 patients had significant eosinophilia, 5 had atypical lymphocytosis, and two had leucopenia. All patients had transaminitis, most at least 10x normal. Peak ALT was 1172IU/L in one patient. None suffered liver failure. One patient developed drowsiness and persistent rotatory nystagmus. Another patient had myositis.

3 patients were tested for HHV6; only one was positive. None had acute EBV infection or reactivation.

All patients were treated with systemic corticosteroids. Doses ranged from 0.3mg/kg/day to 1.6mg/kg/day prednisolone equivalent. Most patients were weaned off steroids by 2 months. 5 patients had worsening symptoms despite oral steroids, with 2 patients requiring readmission. There were no fatalities. One patient developed TRAb+ hyperthyroidism 6 months later.

## Conclusion

DRESS is a rare condition, and diagnosis may also be dependent on physicians’ awareness as 5 of our cases were diagnosed in the past 3 years. Liver involvement is significant, and all cases responded to systemic steroids. However the dose and duration of steroids were not standardized.

